# North Carolina Veterinary Medical Association

**Published:** 1897-03

**Authors:** J. W. Petty

**Affiliations:** Secretary


### NORTH CAROLINA VETERINARY MEDICAL ASSOCIATION.

On December 29, 1896, pursuant to a call by Dr. J. W. Petty, the veterinarians of North Carolina met in the city of Raleigh for the purpose of organizing an association, of which each of the following were declared to be charter members: Drs. C. B. Ellis, Charlotte; T. B Carroll, Wilmington; J. W. Petty, Greensboro; H. G. Bessent, Durham; W. C. McMackin, Raleigh; H. F. Bauer, Greensboro; H. B. Murray, Hickory; J. W. Rollins, Asheville; Daniel Quinlivan, Wilmington; Ashcraft, Monroe. The following officers were elected: President, C. R. Ellis; Vice-President, T. B. Carroll; Secretary and Treasurer, J. W. Petty.

The President appointed the following committees: Constitution and By-laws, Drs. J. W. Petty and H. F. Bauer; On Legislation, W. C. McMackin.

J. W. PETTY,
Secretary,

